# Effort minimization and synergistic muscle recruitment for three-dimensional force generation

**DOI:** 10.3389/fncom.2013.00186

**Published:** 2013-12-20

**Authors:** Daniele Borzelli, Denise J. Berger, Dinesh K. Pai, Andrea d'Avella

**Affiliations:** ^1^Laboratory of Neuromotor Physiology, Santa Lucia FoundationRome, Italy; ^2^Department of Computer Science, University of British ColumbiaVancouver, BC, Canada

**Keywords:** muscle synergies, isometric force, directional tuning, effort minimization, non-negative matrix factorization

## Abstract

To generate a force at the hand in a given spatial direction and with a given magnitude the central nervous system (CNS) has to coordinate the recruitment of many muscles. Because of the redundancy in the musculoskeletal system, the CNS can choose one of infinitely many possible muscle activation patterns which generate the same force. What strategies and constraints underlie such selection is an open issue. The CNS might optimize a performance criterion, such as accuracy or effort. Moreover, the CNS might simplify the solution by constraining it to be a combination of a few muscle synergies, coordinated recruitment of groups of muscles. We tested whether the CNS generates forces by minimum effort recruitment of either individual muscles or muscle synergies. We compared the activation of arm muscles observed during the generation of isometric forces at the hand across multiple three-dimensional force targets with the activation predicted by either minimizing the sum of squared muscle activations or the sum of squared synergy activations. Muscle synergies were identified from the recorded muscle pattern using non-negative matrix factorization. To perform both optimizations we assumed a linear relationship between rectified and filtered electromyographic (EMG) signal which we estimated using multiple linear regressions. We found that the minimum effort recruitment of synergies predicted the observed muscle patterns better than the minimum effort recruitment of individual muscles. However, both predictions had errors much larger than the reconstruction error obtained by the synergies, suggesting that the CNS generates three-dimensional forces by sub-optimal recruitment of muscle synergies.

## Introduction

Object manipulation and tool use require accurate control of the three-dimensional force generated at the hand by the contraction of arm muscles. To generate a force at the hand in a given spatial direction and with a given magnitude, the central nervous system (CNS) has to coordinate the recruitment of many muscles. A desired force vector must results from the sum of the force vectors generated by the contraction of each individual muscle. Thus, the control policy implemented by the CNS must select an appropriate muscle activation pattern for each desired force vector output. Such a mapping from force targets to muscle patterns is the inverse of the biomechanical transformation of muscle contraction into output force. However, because of the redundancy of the muscular apparatus, the solution is not unique and infinitely many muscle patterns can generate the same force output. These patterns only differ with respect to the amount of muscle co-contraction, i.e., the part of the muscle contraction which generates force components that cancel each other (Valero-Cuevas, 2009).

How the CNS coordinates many redundant muscles is a long standing question in motor neuroscience (Bernstein, 1967). One possibility is that CNS selects the muscle pattern for a specific goal by minimizing some cost, such as effort or inaccuracy (Harris and Wolpert, 1998; Fagg et al., 2002; Todorov and Jordan, 2002; Franklin et al., 2008; Kutch et al., 2008). Such minimization may be performed searching among all possible muscle patterns and potentially achieving the global minimum of the cost function. As optimization becomes computationally challenging when it involves a large number of variables, the CNS might search for a solution only within the subset of all possible patterns generated by the combination of a small number of muscle synergies, coordinated recruitment of groups of muscles with specific activation balances or profiles (Tresch et al., 1999; Saltiel et al., 2001; d'Avella et al., 2003; Ting and McKay, 2007; Bizzi et al., 2008; Lacquaniti et al., 2012; d'Avella and Lacquaniti, 2013). However, by reducing the number of variables, i.e., constraining the solution to combinations of muscle synergies, only a value of the cost function generally larger than the global minimum can be achieved. Thus, there is a trade-off between optimality and computational complexity in the solution of the coordination problem.

Whether muscle synergies are a simplifying control strategy actually implemented by the CNS or they represent a parsimonious description of the regularities in the motor output generated by a non-synergistic controller and due to specific task constraints is a debated issue (Kutch et al., 2008; Tresch and Jarc, 2009; Valero-Cuevas et al., 2009; d'Avella and Pai, 2010; Kutch and Valero-Cuevas, 2012; Berger et al., 2013; Bizzi and Cheung, 2013). Evidence for muscle synergies as neural control strategies has come from the observation of low-dimensionality in the muscle patterns. In many species and behaviors the muscle patterns recorded in a variety of conditions can be reconstructed by a combination of a small number of muscle synergies (Tresch et al., 1999; d'Avella et al., 2003, 2006; Ivanenko et al., 2004; Ting and Macpherson, 2005; Torres-Oviedo and Ting, 2007; Overduin et al., 2008; Dominici et al., 2011; Delis et al., 2013). Moreover, neural recordings and stimulation responses suggest that muscle synergies are encoded in the CNS (Saltiel et al., 2001; Ethier et al., 2006; Gentner and Classen, 2006; Hart and Giszter, 2010; Overduin et al., 2012). However, recent simulation studies have argued that the low-dimensionality that might be observed in the muscle patterns during isometric force generation could derive from biomechanical constraints (Kutch and Valero-Cuevas, 2012) and that the shape of the covariance of the force fluctuations recorded during static isometric force production is not compatible with muscle synergies (Kutch et al., 2008).

The aim of this study is to test whether the control policy employed by the CNS for the generation of force minimize effort by either independent recruitment of individual muscles or by synergistic recruitment. We have performed a comparison between the activation of several muscles acting on the shoulder and elbow joints observed during the generation of static isometric force at the hand across multiple three-dimensional force targets and the muscle activation predicted by minimizing effort either over the set of all possible muscle patterns or within the subset of muscle patterns generated by combinations of muscle synergies. To derive these predictions, we have estimated the isometric force generated by each muscle, assuming a linear relationship between rectified and filtered electromyographic (EMG) signal and force, and we have identified time-invariant muscle synergies by non-negative matrix factorization (NMF) (Lee and Seung, 2001; Tresch et al., 2006). While the observed muscle patterns could be reconstructed accurately by the combination of a small number of muscle synergies, they were not well predicted by either minimum effort recruitment of individual muscles or synergies. However, the synergistic prediction had a significantly lower error than the prediction based on individual muscles.

## Materials and methods

### Participants

Nine right handed subjects (5 males and 4 females, mean age 29.6 ± 4.4 years, age range 24–39) participated in the experiment after giving written informed consent. All procedures were approved by the Ethical Review Board of the Santa Lucia Foundation.

### Experimental apparatus and data acquisition

**Figure 1:**
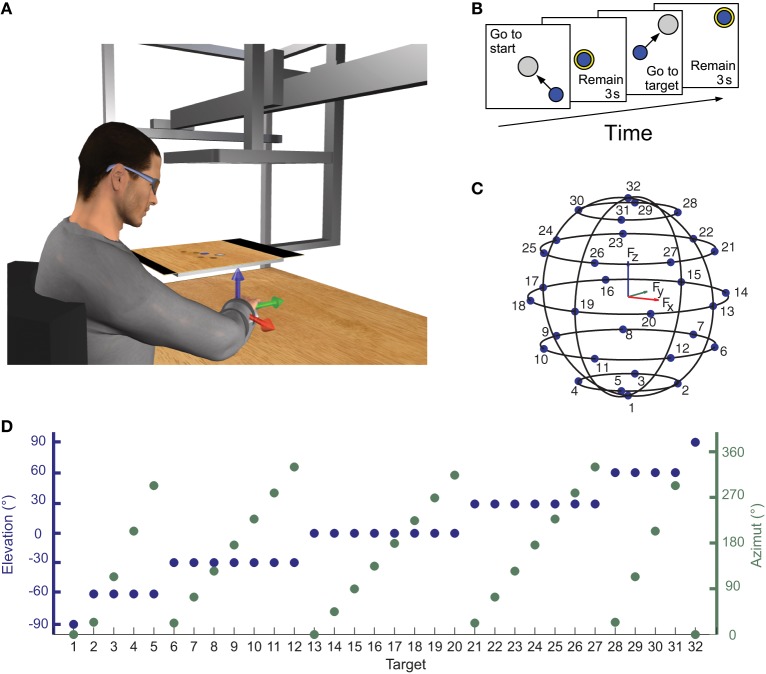
**Experimental apparatus and protocol for measuring isometric force and electromyographic signal. (A)** Subjects sat in front of a desktop with their right arm, wrist, and forearm immobilized in a splint rigidly coupled to a force transducer mounted below the desktop. A mirror occluded subjects' view of their hand and reflected a virtual scene displayed by a flat horizontal monitor placed at subjects' eyes height and matching the real desktop view. A spherical cursor was displayed at the center of the palm when no forces were applied on the splint. Cursor motion was simulated in real time as a mass accelerated by the force applied by the subject on the splint, a viscous force, and an elastic force proportional to the distance for the rest position. **(B)** Subjects were instructed to perform center-out reaching trials in which they had to maintain the cursor in a central start location for 3 s, reach a target, and maintain the cursor at the target location for 3 s. After this time the subject relaxes to return to the starting position and be ready to start a new trial. **(C,D)** Force targets were distributed on the surface of a sphere of radius of 20% MVF, arranged on horizontal planes at different heights. The elevation and the azimuth angles of each one of the 32 targets were chosen to distribute the targets approximately uniformly on the sphere surface.

Subjects sat on a racing car seat with their torso immobilized by safety belts anchored behind their shoulders and hips. They inserted their right hand and forearm in a splint that immobilized hand, wrist, and forearm positioned on a desktop in front of them. The splint was attached to a steel bar and mechanically connected via a steel rod to a 6-axis force transducer (Delta F/T Sensor, ATI Industrial Automation, Apex, NC, USA) mounted below the desktop. In this posture the center of the palm was aligned with the body midline at the height of the sternum and the elbow was flexed approximately by 90°. The height of the desktop and the distance of the chair from the desktop could be adjusted according to the subject's size. The subject view of his right hand was occluded by a mirror (29.7 × 21 cm), parallel to the desktop, that reflect the image displayed by a 21-inch LCD monitor (Syncmaster 2233, Samsung Electronics Italia S.p.A., Cernusco sul Naviglio, MI, Italy), also parallel to the desktop (Figure 1A). The height of the monitor was adjusted at the height of the subjects' eyes and the mirror was positioned halfway between the subjects' hand and the monitor. During the experiments subjects wore 3D shutter glasses (3D Vision P854, NVIDIA Corporation, Santa Clara, CA, USA) and viewed stereoscopically a virtual desktop matching the real desktop and a spherical cursor positioned, at rest, approximately at the center of the palm. The virtual scene was rendered by a 3D graphics card (Quadro Fx 3800, NVIDIA) on a PC workstation using custom software. Force targets were shown as transparent gray spheres and force feedback was provided by the displacement of the spherical blue cursor (Figure 1B). The scene was updated at 60 Hz with the cursor position processed by a second dedicated data-acquisition PC workstation running a real-time operating system and transmitted to the first workstation through an Ethernet link using the UDP protocol. Cursor motion was simulated in real time as a mass accelerated by the force applied by the subject on the splint, a viscous force, and an elastic force proportional to the distance from the rest position. The spring constant was set such that the force applied to maintain the cursor stationary at the target, distant 5 cm from the center of the palm, had a magnitude equal to 20% of the subject's mean maximum voluntary force (MVF) across force directions (see below). To maintain fast responses to changes in force while reducing the effect transducer noise when the force was stationary, the mass was adjusted adaptively in the range 15–140 g as a sigmoidal function of the rate of change in the magnitude of the recorded force. The damping constant was set to make the system critically damped.

Electromyographic activity from 17 muscles acting on the right shoulder and elbow was recorded with active bipolar electrodes (DE 2.1, Delsys Inc., Boston, MA), after band-pass filtering (20–450 Hz) and amplification (gain 1000, Bagnoli-16, Delsys Inc.). The following muscles were recorded: teres major (TeresMaj), infraspinatus (InfraSp), latissimus dorsi (LatDors), inferior trapezius (TrapInf), middle trapezius (TrapMid), superior trapezius (TrapSup), brachioradialis (BracRad), biceps brachii, long head (BicLong), biceps brachii, short head (BicShort), triceps brachii, lateral head (TriLat), triceps brachii, long head (TriLong), triceps brachii, medial head (TriMed), anterior deltoid (DeltA), middle deltoid (DeltM), posterior deltoid (DeltP), pectoralis major clavicular (PectClav), pectoralis major sternal (PectStern). Correct electrode placement was verified by observing the activation of each muscle during specific maneuvers. Force and EMG data were digitalized at 1 kHz using an A/D PCI board (PCI-6229, National Instrument, Austin, TX, USA). Only the forces (F_x_ lateral direction on the horizontal plane, positive to the right; F_y_ frontal direction on the horizontal plane, positive away from the chest; F_z_ vertical direction, positive up) were used during the experiment.

### Experimental protocol

For each subject, the MVF along the direction of the 20 vertices of a dodecahedron was estimated at the beginning of the experiment and used to scale the magnitude of the force targets. For each direction the maximum force magnitude was recorded in two trials in which subjects were instructed to generate maximum force in a spatial direction indicated by an arrow. Subjects then performed a series of 160 trials generating forces in 32 directions (5 series of trials in all directions). The target directions were chosen to be approximately uniformly distributed on the surface of a sphere with radius of 0.2 MVF. Targets were arranged on horizontal planes at different heights. On the F_z_ = 0 plane, 8 targets were equally distributed on a circumference. The height of the other horizontal force planes was calculated such that the difference in elevation angle (ϕ = tan^−1^(F_z_/(F^2^_x_ + F^2^_y_)^1/2^)) of two adjacent planes was approximately equal to the angle between two adjacent targets of the F_z_ = 0 plane. The number of targets on each plane was chosen such that the azimuth angle (ϑ = tan^−1^(F_y_/F_x_)) difference between two adjacent targets on the plane was as close as possible to the angle between two targets on the F_z_ = 0 plane (45° for 8 targets, see Figures 1C,D). At the beginning of each trial subjects were instructed not to apply any force and to maintain the cursor for 3 s (rest phase) within a transparent yellow sphere with a radius larger than the cursor sphere radius by 2% MVF and aligned with the center of the palm. A target, indicated by a gray transparent sphere with a radius larger than the cursor sphere radius by 2% MVF was then displayed in one of the 32 locations and subjects were instructed to move the cursor to the target by applying force (Figure 1B). The target sphere turned yellow when the cursor was inside it. Finally, subjects were required to maintain the cursor within the target for 3 s (hold phase) to successfully end the trial.

### Data analysis

EMG data were used to characterize the directional tuning of muscle activations, to identify time-invariant muscle synergies, and, together with force data, to estimate an EMG-to-force matrix. One subject was excluded from the analysis after realizing that during the experiment the position of the cursor when the subject was not applying any force to the splint (at the beginning of each trial) had drifted, likely due to a lack of proper immobilization of the hand and forearm in the splint. A few trials in which the remaining eight subjects were not able to reach or remain in the target (3.4 ± 4.5 over 160 total trials, mean ± *SD*, range 0–13) as well as a few additional trials with EMG artifacts (6.0 ± 4.2, range 1–13) were excluded from the analysis. Finally, a few trials of the MVF block with EMG artifacts were also excluded from the analysis. The total number of excluded trials was 15.7 ± 12.3, range 1–33.

#### Directional tuning of muscle activations

EMG data were rectified and digitally low-pass filtered (2nd order Butterworth, 5 Hz cutoff) and re-sampled at 100 Hz to reduce data size. In each trial, mean EMG activity of each muscle during the last 0.6 s of the rest phase was used to estimate the baseline noise level of each muscle which was then subtracted from the rest of the data. Filtered EMG waveforms for each muscle were aligned to the beginning of the hold phase and then averaged across repetitions of the same target to construct directional tuning curves. Averaged EMG for each muscle were normalized to the maximum voluntary contraction across direction (MVC) recorded during MVF.

The directional tuning of each muscle activation was also fitted by a spatial cosine function:

$$\begin{array}{l} m(f;f_{PD})=f^{T}f_{PD}+m_{offset}=f_{PD}[\cos\varphi \cos\varphi_{PD}\\ \;+\sin\varphi \sin\varphi_{PD}\sin(\vartheta -\vartheta_{PD})]+m_{\text{offset}}, \end{array}$$

where ***f*** in the unit vector pointing in the direction of the force target, ***f_PD_*** is a preferred direction vector with length *f_PD_*, azimuth angle ϑ*_PD_*, and elevation angle ϕ*_PD_*, and *m*_offset_ is an offset level. The parameters of the preferred direction vector and offset were estimated by multiple linear regressions (Matlab function regress) and the significance of the tuning assessed by an *F*-test.

#### Muscle synergies

Muscle synergies were identified by a NMF algorithm (Lee and Seung, 1999, 2001). Muscle activation vectors (**m*^k^***) constructed with the rectified, filtered, and averaged EMG waveforms of each muscle during the hold phase of *k*-th trial, normalized to MVF after baseline noise level subtraction. Each vector (matrix column) was reconstructed as the combination of a unique set of *N* time-invariant synergies (**w***_i_*) scaled by time-varying synergy activation coefficients (*c^k^_i_*)

$$m^{k}\;=\sum_{i=\text{1}}^{N}c_{i}^{k}w_{i}$$

or, equivalently, in matrix notation, **M** = **W C**. For each *N* from 1 to the number of muscles, the extraction algorithm was repeated 10 times and the repetition with highest reconstruction *R*^2^ was retained. *R*^2^, the fraction of total variation explained by the synergy model, was defined as 1 - SSE/SST, where SSE is the sum of the squared residuals and SST is the sum of the squared differences between the recorded muscle patterns and their mean.

The number of synergies *N* is a free parameter that we chose as the smallest number that reconstructed accurately the data variation taking noise and the directional tuning of synergy activation coefficient into account. In previous studies using decomposition algorithms to identify muscle synergies, *N* was selected to capture the structured data variation not due to noise either according to a threshold in *R*^2^ (Tresch et al., 1999; Ting and Macpherson, 2005; Torres-Oviedo et al., 2006) or by identifying a change in slope in the *R*^2^ curve (Cheung et al., 2005; d'Avella et al., 2006; Tresch et al., 2006). We considered both criteria, and we computed (i) the smallest *N* for which the *R*^2^ was larger than 0.9 and (ii) as the point at which the *R*^2^ vs. *N* curve had a change in slope (MSE error of linear fit from *N* to 17, the number of muscles, below 10^−4^). In case of mismatch between the number of synergies selected according to the two criteria, we chose the one set of synergies with a more uniform directional distribution of preferred directions of the synergy activation coefficients (the direction of the maximum of the cosine function best fitting the directional tuning). To do so, for each one of the two synergy sets, we arranged their preferred direction vectors on a unit sphere, we considered all pairs, and we selected the set of synergies with the smallest number of pairs with an angular difference below 20°. Finally, the elements of each synergy vector (**w***_i_*) in the selected set were normalized to their maximum value.

Directional tuning curves for the synergy activation coefficients, as for the muscle activations, were constructed by averaging their values in the hold phase and across trials to the same target.

#### EMG-to-force matrix

The isometric end-point force (**f**) generated at the hand with the arm in a fixed posture (as both the trunk and the forearm were immobilized) by a muscle activation pattern (**m**) was modeled as linear combination of the end-point force associated to each muscle, **f** = **H m**, where **H** is a matrix with dimensions [3 × *N_m_*] (*N*_m_ number of muscles). For each subject we estimated such matrix using multiple linear regressions of each force component, low-pass filtered (2nd order Butterworth, 5 Hz cutoff) with the rectified, filtered, re-sampled, baseline subtracted, MVC normalized EMG data recorded during the hold phase in all conditions. While the relationship between muscle activation and end-point force is generally not linear, for low muscle activation required for the force magnitude of the targets used in the experiment (0.2 MVF) linearity provided an adequate approximation (Lawrence and De Luca, 1983).

#### Minimum effort predictions

We predicted the observed muscle activation pattern (**m**^obs^) for each force target either by minimizing the sum of squared muscle activation (**m**^musc^) (Buchanan and Shreeve, 1996; van Bolhuis and Gielen, 1999; Todorov and Jordan, 2002) or by minimizing the sum of squared synergy activations (**m**^syn^), under the constraint that the predicted pattern generates the desired force target according to the linear EMG-to-force mapping (**H**):

$$m^{\text{musc}}=\arg\min({\left\|m\right\|}^{2})\text{ such that }f=Hm$$

$$\{\begin{array}{c} m^{\text{syn}}=Wc^{\text{syn}}\\ c^{\text{syn}}=\arg\min({\left\|c\right\|}^{2})\text{ such that }f=HWc \end{array}$$

We used the MATLAB function quadprog to find these minima.

#### Minimum effort prediction with random muscle synergies

We performed a Monte Carlo simulation to assess the significance of the prediction obtained by minimizing synergy effort. We compared the mean squared residual of the minimum synergy effort prediction with the distribution of the mean squared residuals obtained with 200 sets of random synergies. For each subject, after randomly shuffling over the columns (trials) each row (muscle) of the muscle activation data matrix (**M**), random synergies were generated either selecting a number of columns equal to the number of synergies identified from the data or by extracting the same number of synergies from the shuffled data matrix.

#### Statistical analysis

A Wilcoxon rank-sum test was performed for each subject to evaluate if the mean over force targets of the squared error of the prediction obtained minimizing muscle effort (**m**^musc^), (ε^musc^)^2^ = ||**m**^musc^ – **m**^obs^||^2^, was statistically different from the squared error of the prediction obtained minimizing synergy effort (**m**^syn^), (ε^syn^)^2^ = ||**m**^syn^ – **m**^obs^||^2^.

## Results

**Figure 2:**
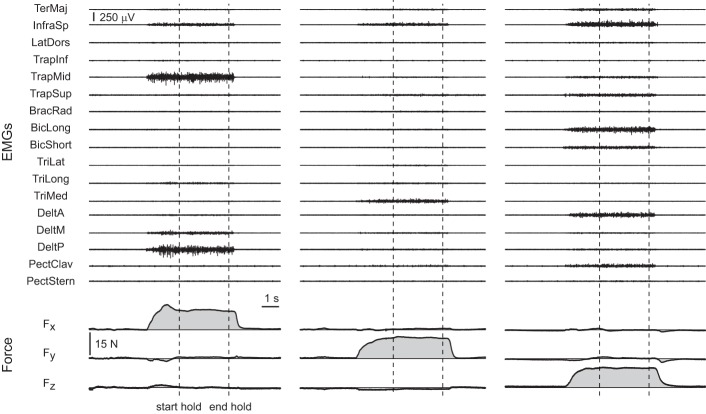
**Examples of raw EMG and force data**. Data were recorded during three trials of subject 8 with targets at 20% MVF along the positive F_x_ axis (*first column*, target 16 in Figure 1), the positive F_y_ axis (*second column*, target 18 in Figure 1), and the positive F_z_ axis (*third column*, target 32 in Figure 1). The vertical dashed lines indicate the beginning and the end of the hold phase.

All subjects were able to reach the force targets and to maintain the force within the required 2% MVF tolerance for 3 s. Examples of the raw EMG and force data recorded for three trials to targets are shown along the positive F_x_, F_y_, and F_z_ axes in Figure 2.

### Directional tuning of muscle activations

**Figure 3:**
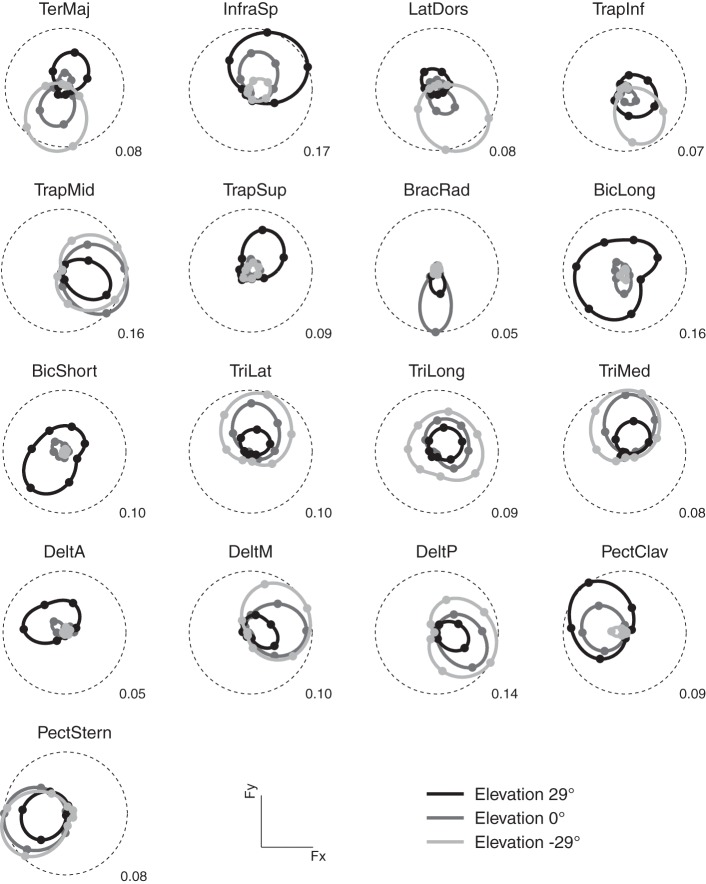
**Example of directional tuning of muscle activations**. Polar plots representing the average EMG activity (hold phase, normalized to MVC) for targets on three horizontal planes at different elevations (*light gray*: −29°, targets 6–12, *medium gray*: 0°, targets 13–20, *dark gray*: 29°, targets 21–27) recorded in subject 8. Numerical value at the bottom right of each plot represents the fraction of mean MVC across all directions for each muscle and corresponds to the radius of the dashed circle. The direction of each marker represents the direction of the horizontal force components, its radius the average EMG activity when holding the target in that direction. Markers are interpolated by splines in polar coordinates.

As in previous studies (Flanders and Soechting, 1990; Roh et al., 2012), we found that the activation of most muscles was modulated by force direction. Figure 3 illustrates the modulation of the activity of 17 arm muscles recorded in subject 8 as a function of the azimuth of the force target on three different horizontal planes (elevation angles: −29, 0, 29°). For each muscle and target elevation, the directional tuning of the mean activity during the hold phase is illustrated by a polar plot in which the muscle activity is indicated by the radial distance of a marker in the direction of the target azimuth. Most muscles showed a directional tuning resembling the tuning expected by a spatial cosine function. For muscles with a preferred direction vector of the best fitting spatial cosine function lying close to the horizontal plane (e.g., TrapMid and PectStern), their azimuth directional tuning resembles a circle tangent to the origin. For muscles with a large vertical component in their preferred direction (e.g., BicLong, BicShort, TriLong, TriLong, and TriMed), the dependence of their activation on elevation is evident in the different radii of the circles. One muscles (BracRad) had a very narrow and non-significant spatial cosine tuning (*p* = 0.14). Other muscles had a significant (*p* < 0.05) but poor (*R*^2^ value of the cosine fit less than 0.5) spatial cosine tuning (TeresMaj, LatDors, TrapInf, and TrapSup). Across subjects, 0.9 ± 0.3 (mean ± SD) muscles had a non-significant (*p* > 0.05) spatial cosine tuning and 3.2 ± 1.8 a poor fit (*R*^2^ < 0.5).

### Muscle synergies

**Figure 4:**
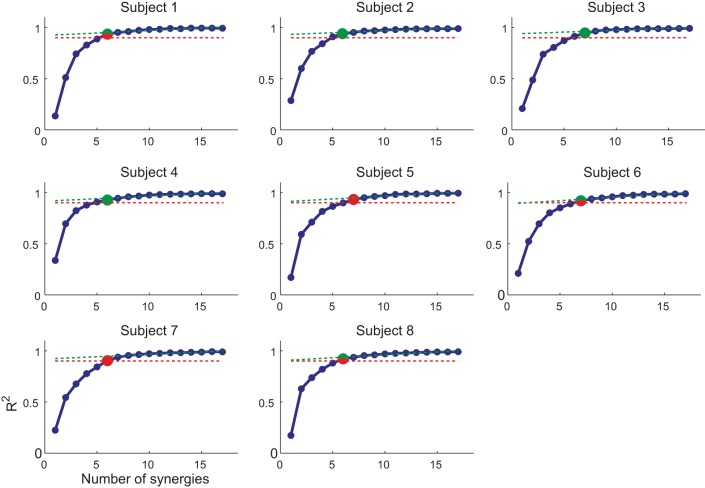
**Selection of number of synergies**. The number of synergies (N) is chosen for each subject as (i) the smallest *N* for which the *R*^2^ value (*blue markers and line*) was larger than 0.9 (*red dashed line*) or (ii) the point at which the *R*^2^ vs. *N* curve had a change in slope [MSE of linear fit from *N* to max(*N*) below 10^−4^, *green dashed line*]. In case of mismatch between the two criteria, the set of synergies with smallest number of similar preferred directions was selected (*red/green marker*, smallest number of synergy pairs with an angular difference between preferred direction below 20°).

**Figure 5:**
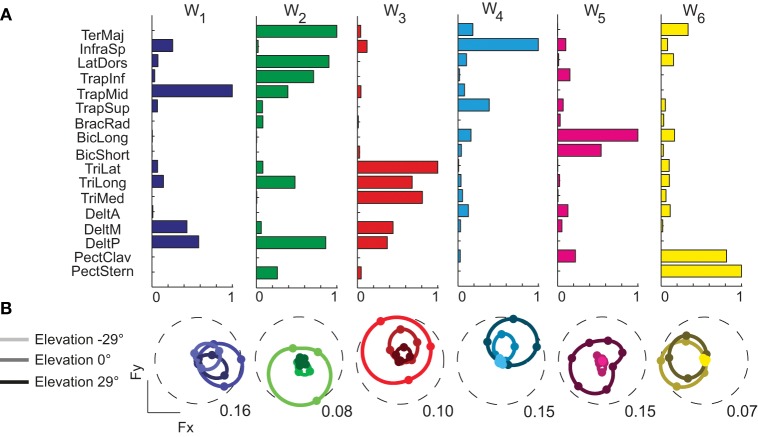
**Example of muscle synergies and directional tuning of activation coefficients. (A)** Six synergies (W_1_–W_6_) identified by NMF from the filtered and time-averaged EMGs of subject 8 recorded during the hold phase of all trials. The bar plot in each column (color coded) shows the components of one synergy vector, normalized to its maximum. **(B)** Directional tuning (polar plot as in Figure 3) of the synergy activation coefficients for force targets on three horizontal planes.

We decomposed the muscle patterns recorded during the hold phase as combinations of muscle synergies identified by the NMF algorithm. Across subjects (Figure 4) the number of synergies selected according to a threshold either in the fraction of the total data variation explained by the synergies (*R*^2^) or in the mean squared error of a linear fit of the final portion of the *R*^2^ curve (see Materials and Methods) ranged from 6 to 7 (6.4 ± 0.5, mean ± SD). The corresponding *R*^2^ values ranged from 0.90 to 0.95 (0.93 ± 0.01). Thus, a small number of synergies captured the modulation of activity in many arm muscles across directions and magnitudes of isometric force generated at the hand. Figure 5A shows the six synergies identified in the muscle patterns of subject 8. Each synergy has a different balance of activation across muscles, with some muscles more strongly active than others (TrapMid, DeltM, and DeltP in W_1_, TerMaj, LatDors, TrapInf, TrapMid, TriLong, DeltP, and PectMajStern in W_2_, TriLat, TriLong, TriMed, DeltM and DeltP in W_3_, InfraSp and TrapSup in W_4_, BicLong and BicShort in W_5_, TerMaj, PectStern, and PectClav in W_6_) and with many muscles recruited in multiple synergies.

Synergy activation coefficients were in most cases also well captured by a spatial cosine function. The directional tuning of the activation coefficients of the six synergies of subject 8 (Figure 5B) was always significant (*p* < 0.0001) and well reconstructed by a cosine fit (*R*^2^ > 0.5). Across subjects, only subject 6 had 4 out of 7 synergy activation coefficients not well fitted by a cosine functions (*p* = 0.40, 0.22, 0.05, 0.05) while all other subjects had a significant (*p* < 0.05) spatial cosine tuning. Across all subjects, only 1.2 ± 1.7 synergy activation coefficients had a poor fit (*R*^2^ < 0.5).

### EMG-to-force matrix and synergy directional tuning

**Figure 6:**
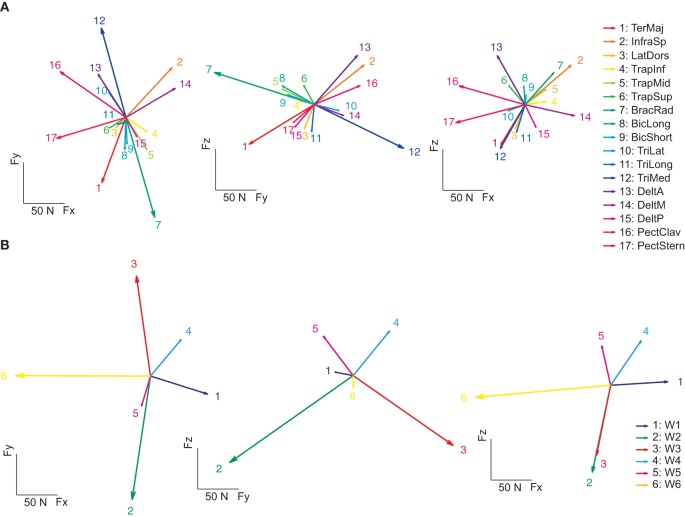
**Example of spatial forces associated to muscle and synergies. (A)** The EMG-to-force matrix ***H*** (*left*: projections of the columns of ***H*** on the F_z_ = 0 plane, *middle*: projection on the F_x_ = 0 plane, *right*: projection on the F_y_ = 0 plane) estimated by linear regression of EMG and force data for subject 8. **(B)** Forces associated to the synergies (columns of the matrix obtained by multiplying the EMG-to-force matrix ***H*** by the synergy matrix ***W***) of subject 8.

As in previous studies of muscle activation during isometric force production (Osu and Gomi, 1999; Valero-Cuevas et al., 2009), we modeled the mapping between EMGs and sub-maximal magnitude (20% MVF) end-point force linearly. An EMG-to-force matrix (**H**) was estimated with multiple linear regressions of the mean EMG and forces recorded in the hold phase for each subject. Figure 6A illustrates force vectors associated to the activation of each muscle (columns of **H**) for subject 8. These force vectors in most cases matched the pulling directions of the muscles expected from their anatomical configuration. For example, on the horizontal plane (*left*), BracRad (elbow flexors) and TeresMaj (shoulder internal rotator and adductor) were associated to dorsally directed (negative F_y_) forces, TriMed (elbow extensors) to a ventrally directed (positive F_y_) force, PectClav and PectStern (shoulder flexors) to medially directed (negative F_x_) forces, and DeltM (shoulder abductor) to a laterally directed (positive F_x_) force. On the sagittal plane (*middle*), DeltA (shoulder adductor), InfraSp (shoulder external rotator), and PectClav showed a large rostral (positive F_z_) and ventral force, BracRad a large rostral and dorsal force, TeresMaj a large caudal (negative F_z_) and dorsal force, and TriMed a large caudal and frontal force. In the frontal plane (*right*) the two portions of pectoralis major showed distinct rostro-caudal (F_z_) components. Across subjects, the forces recorded during the hold phase were reconstructed accurately by the product of the EMG-to-force matrix times the recorded EMGs (*R*^2^ = 0.89 ± 0.02, mean ± *SD*, *n* = 8, for the reconstruction of the individual force samples in all trials; *R*^2^ = 0.97 ± 0.01 for the reconstruction of the force averaged across time and trials to the same target by averaged EMGs).

We also estimated the force associated to the activation of individual muscle synergies by multiplying the EMG-to-force matrix with the synergy matrix (columns of the **HW** matrix, Figure 6B). Each synergy had a distinct force direction in space. W_1_ was associated to a lateral force, W_2_ to a dorso-caudal force, W_3_ to a ventro-caudal force, W_4_ to a ventro-rostral-lateral force, W_5_ to dorso-rostral force, and W_6_ to a medial force. However, with respect to individual muscle forces, there were larger angular differences between individual synergy force directions.

### Muscle activations predicted by minimum effort criteria

**Figure 7:**
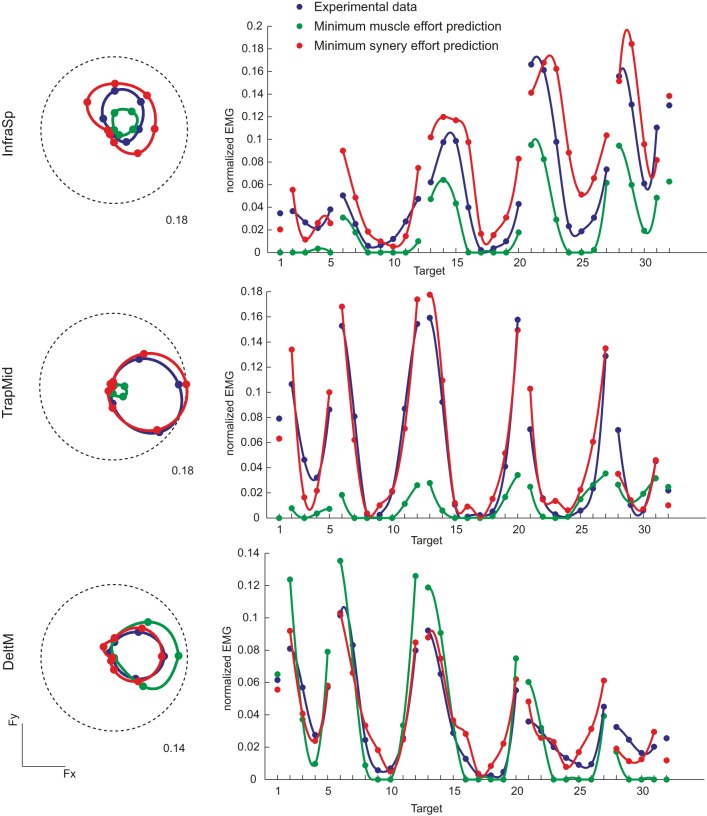
**Examples of directional tuning of muscle activation observed and predicted by minimum effort criteria**. Muscle activations for three muscles of subject 8 are illustrated. **Left:** Polar plots of the directional tuning on the horizontal force plane (F_z_ = 0, targets 13–20). EMG activity, normalized to the MVC value of each muscle, is averaged during the hold phase. Dashed circles represent the normalized activity indicated by the label. **Right:** Average EMG activity for all 32 targets. *Blue* markers and lines (interpolating the markers with spline curves in polar coordinates) represent experimental data, *green* markers and lines (interpolating the markers with spline curves with negative values set to zero) represent predictions according to the linear EMG-to-force model with the minimum muscle effort criterion, *red* markers and lines with the minimum synergy effort criterion.

We compared the muscle activation observed in all force directions with those predicted by minimizing either muscle effort or synergy effort. Examples of the directional tuning curves on the horizontal force plane (polar plot, *left*) and for all directions (*right*) of three muscles (InfraSp, TrapMid, and DeltM) of subject 8 are illustrated in Figure 7. In all three cases the predicted tuning curves peak in same directions as the observed curves but in some cases they do not fit well the whole curve. For InfraSp (*first row*), the minimum muscle effort curve underestimates the observed curve and the minimum synergy effort curve overestimates it. For TrapMid (*second row*), muscle effort minimization predicts a very weak activation while the minimum synergy effort prediction closely matches the observed data. For DeltM (*third row*), the minimum synergy effort prediction again matches the observed data while the minimum muscle effort prediction overestimates them. These differences between the two predictions depend on how the forces associated to the muscles (the columns of the H matrix, Figure 6A) and the synergies (Figure 6B) can be combined to minimize effort. For example, the minimum muscle effort criterion predicts an activation of TrapMid much weaker than the minimum synergy effort criterion because the minimum muscle norm solution is achieved by recruiting more strongly other muscles with a pulling direction close to that of TrapMid but with a larger forcer magnitude (in particular BracRad, see Figure 6A). In contrast, TrapMid has a stronger activation with the minimum synergy norm solution because it is recruited within W_1_ (see Figure 5) and no other synergies can generate forces in the medial-dorsal direction with small activations.

**Figure 8:**
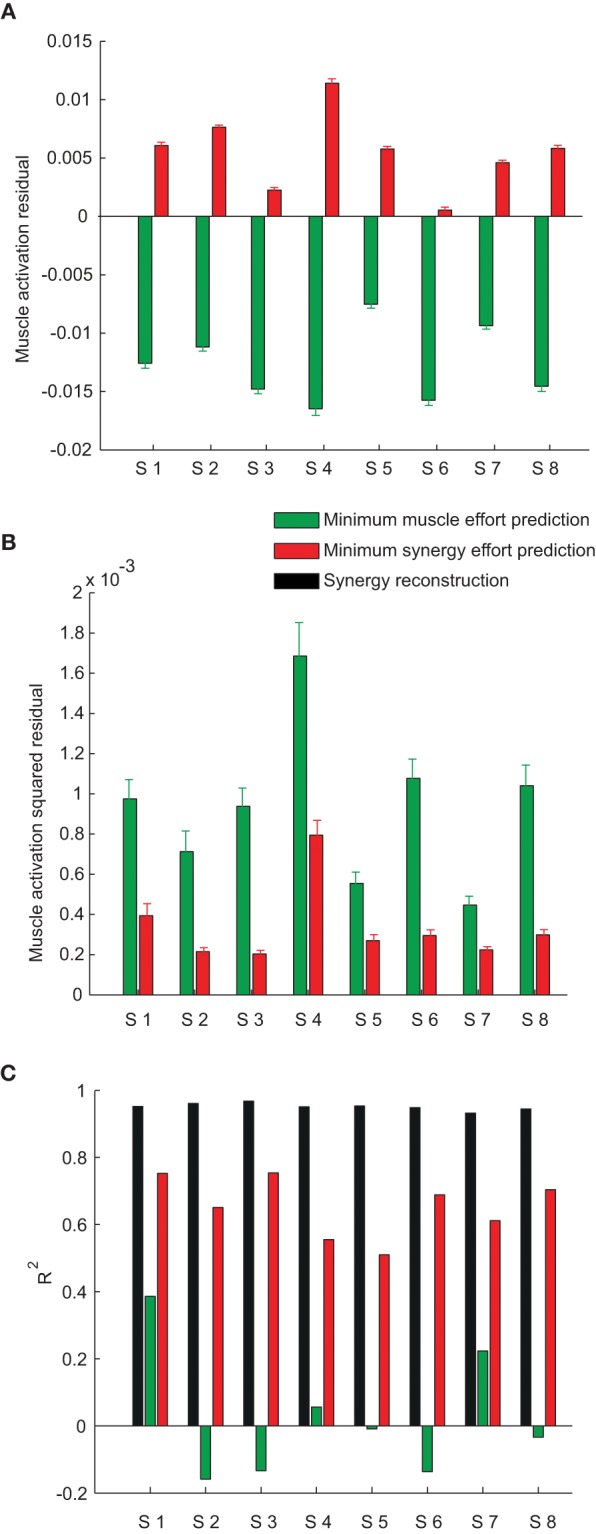
**Model prediction error. (A)** Mean ± SE of the residual, over muscles and force targets, of the minimum muscle effort prediction (green bars) and the minimum synergy effort prediction (red bars) for all subjects. **(B)** Mean ± SE of the squared residual, over muscles and force targets, of the minimum muscle effort prediction (green bars) and the minimum synergy effort prediction (red bars) for all subjects. **(C)** Comparison of the *R*^2^ values for the synergy reconstruction (black bars) and the model predictions.

Across subjects, we noticed that the mean residual of the minimum muscle effort prediction over all muscles and targets was always negative (sign test, *p* < 0.0001 for all subjects, see *green bars* in Figure 8A) and that the mean residual of the minimum synergy effort prediction was always positive (*p* < 0.01 for all subjects except subject 6, *red bars* in Figure 8A). Thus, the minimum muscle effort criterion underestimated the observed muscle activations and the minimum synergy effort criterion overestimated them. The minimum muscle effort underestimation corresponds to a larger than minimal amount of co-contraction in the observed muscle patterns. Indeed, the amount of co-contraction, quantified by the mean Euclidian norm of the projection of the muscle patterns onto the null space of the EMG-to-force matrix, was significantly higher for the observed data than for the minimum muscle effort prediction (sign test, *p* < 0.0001 for all subjects; mean ± *SD* across subjects: 0.16 ± 0.04 for the data and 0.09 ± 0.02 for the prediction). The mean null space norm for the minimum synergy effort criterion (mean ± *SD* across subjects: 0.19 ± 0.04) was higher than the mean norm for the minimum muscle effort criterion but also slightly higher than the mean norm for observed data (sign test, *p* < 0.05 for subjects 2, 4, 5, 7, and 8) possibly due to inaccuracies in the estimation of the EMG-to-force matrix. Finally, we found that the residuals for many muscles were not normally distributed. Across subjects, the residuals of the minimum muscle effort prediction of the activation of individual muscles had a distribution over different targets significantly different from the normal distribution (Lilliefors test, *p* < 0.05) in 62% of cases (84 cases over 17 muscles in 8 subjects) for the minimum muscle effort model and in 31% of cases for the minimum synergy effort model. However, we could not discern any clear pattern in the residuals.

We then compared the prediction error magnitudes. We found that the mean squared residual of the minimum synergy effort prediction was lower than the mean squared residual of the minimum muscle effort prediction (Figure 8B). The difference of the squared residual, averaged across muscles and targets, between the two criteria was significant (Wilcoxon rank-sum test, *p* < 0.0001, *n* = 8). To assess the significance of these results we compared, for each subject, the mean squared residual of the minimum synergy effort prediction with the distribution of the mean squared residual obtained applying the minimum effort criterion on random synergies. We performed a Monte Carlo simulation, generating, for each subject, random synergies either randomly shuffling the EMG data or performing NMF on randomly shuffled EMG data. We found that the mean squared residual obtained with the synergies extracted from the data was much smaller than the residuals obtained with both types of random synergies in all subjects (empirical *p* < 0.005), indicating that the value of the mean squared residual of the minimum synergy effort prediction was not simply due to the small number of synergies but depended on the actual structure of the synergies.

Finally, we compared the squared prediction error of the two models with the reconstruction error of the synergies. For all subjects, the fraction of the variation of the muscle patterns across force targets, averaged over repetitions to the same target, explained by the combinations of the synergies (Figure 8B, *black bars*, *R*^2^ = 0.95 ± 0.01, mean ± SD) was much higher than the fraction explained by both models. However, the minimum muscle effort model had a smaller *R*^2^ value (*green bars*, 0.02 ± 0.20) than the minimum synergy effort model (*red bars*, 0.65 ± 0.10).

## Discussion

We investigated muscle patterns underlying the generation of isometric force at the hand along 32 uniformly distributed directions in tri-dimensional space. Across subjects, the directional tuning of most muscles was well captured by a spatial cosine function and muscle patterns for all force targets could be reconstructed by the combinations of 6 or 7 muscle synergies identified by NMF. We then estimated the force associated to muscle activation by multiple linear regressions and we used such linear mapping to predict the minimum muscle effort and the minimum synergy effort muscle patterns for each force target. We found that the prediction error with both minimum effort criteria was larger than the synergy reconstruction error but the error obtained minimizing the synergy effort was significantly smaller than the error obtained minimizing muscular effort. These results suggest that the CNS recruits sub-optimal combinations of muscle synergies to generate isometric forces.

The estimation of the mapping between muscle activity and isometric force at the hand was necessary to predict the minimum effort muscle patterns for a given force target. We approximated such mapping during the generation of a static isometric force (hold phase) as a linear transformation between rectified, low-pass filtered, MVC-normalized EMGs and low-pass filtered forces. We could then estimate an EMG-to-force matrix by linear regression of the force components as a function of the activity of all recorded muscles. The assumption of linearity is reasonable when the posture does not change and generated forces are much smaller than the MVF (Lawrence and De Luca, 1983), as in our case. Linear models have been used before to predict isometric forces from EMG recordings (Valero-Cuevas et al., 2009) and minimum effort muscle patterns (Fagg et al., 2002). However, our linear approximation of the mapping between muscle activity and force may have contributed to the model prediction error. Qualitatively the muscle pulling directions estimated by multiple linear regressions appeared compatible with the directions expected from the known anatomical arrangement and mechanical action of the muscles. A quantitative evaluation of the EMG-to-force matrix obtained with our simple procedure might be possible by comparing such matrix with one derived using a detailed musculoskeletal model of the arm (Holzbaur et al., 2005) but such comparison is challenging because of the many subject-specific anatomical and physiological parameters that need to be determined in order to generate reliable predictions with a musculoskeletal model. Thus, we believe that our simplifying assumptions are adequate for the purpose of comparing the two minimum effort criteria, since both minimizations rely on the same EMG-to-force matrix.

A second concern with our approach is the selection of the number of synergies. We used two criteria frequently used in the muscle synergy literature (Tresch et al., 2006; Delis et al., 2013): the total variation accounted by the synergies (synergy reconstruction *R*^2^) (Tresch et al., 1999; Torres-Oviedo et al., 2006) and the detection of a change in slope in the *R*^2^curve (d'Avella et al., 2003; Cheung et al., 2005). In case of discrepancy between the two criteria we selected the number of synergies with a more uniform distribution of the preferred directions of the synergy activation coefficients. Both criteria depend, however, on *ad-hoc* thresholds and thus, while they ensure a meaningful comparison across subjects, they cannot guarantee that the correct number of synergies is selected. In a recent study of the muscle synergies underlying force production in a task similar to ours (Roh et al., 2012), a smaller number of synergies has been reported (3–5). Such difference may be due to the smaller number of muscles recorded in that study (8 vs. 17 in ours) and to the different definition of variance accounted for (VAF). As muscle patterns are multidimensional observations, we referred the synergy reconstruction error to the total variation (Mardia et al., 1979) of the muscle patterns, i.e., the multidimensional generalization of the variance of a scalar observation, and we defined *R*^2^ = 1 − SSE/SST, with SSE the sum of the squared residual and SST as the sum of the squared residual with respect to the mean muscle pattern, proportional to the total variation (d'Avella et al., 2006; Delis et al., 2013). Roh and colleagues, in contrast, defined VAF = 100 × (1 − SSE/SST), with SST sum of the squared data, i.e., without subtracting the mean muscle pattern. As a consequence such VAF value is higher than the *R*^2^ value for the same number of synergies and a smaller number of synergies are selected with the same threshold (90%). When we performed the same analysis of Roh and collaborators on our data, using the same 8 muscles, we found a comparable number of synergies (3–5). Notably, a minimum number of 4 synergies is required to generate forces in all spatial directions by non-negative combinations (Davis, 1954).

A number of previous studies have investigated whether the observed muscle patterns can be the result of effort minimization. Buchanan and Shreeve (1996) used models of the muscles about the elbow (11 muscles) and wrist (5 muscles) to compare the observed directional dependence of muscle activation with the prediction from the minimization of several cost functions, including sum of muscle force, stress, and normalized force (Buchanan and Shreeve, 1996). The choice of cost function had little influence on the results and all cost functions were not able to reliably estimate muscle activation as a function of force direction, even if predictions at the wrist were more favorable than those at the elbow due to the smaller number of muscles and degrees-of-freedom. A sensitivity analysis indicated that the discrepancies between predicted and observed values could not be explained by errors in the physiological parameters of the models, calling into question the applicability of optimization analysis to study such tasks. Our results are in accordance with those observations and extend them to the generation of hand forces by a larger number of muscles acting at the elbow and the shoulder. Moreover, the larger prediction errors that we obtained minimizing muscle effort with respect to synergy effort suggest that the synergistic recruitment of muscles contributes to the sub-optimal co-activation of muscles.

Investigating wrist movements, Fagg and colleagues showed that minimizing effort, defined as we did as the sum of squared muscle activations, yields muscle activation patterns qualitatively similar to those observed experimentally, in particular, reproducing the observed cosine-like recruitment of the muscles as a function of movement direction and also appropriately predicting that certain muscles will be recruited more strongly in movement directions that differs significantly from their direction of action (Fagg et al., 2002). While our model predictions also reproduced cosine-like recruitments and qualitatively similar directional tuning curves in several muscles, in many cases we did observed qualitative and substantial discrepancies between predicted and observed muscle activations. Such poorer model performance may be due, as observed by Buchanan and Shreeve, by the larger number of muscles and degrees-of-freedom considered in our study.

A recent study used a static quadrupedal musculoskeletal model of the cat to predict limb forces and muscle activity in response to multidirectional postural perturbations while minimizing different formulations of control effort, including muscle and synergy effort (McKay and Ting, 2012). Patterns of muscle activity producing forces and moments at the center of mass necessary to maintain balance and the resulting ground reaction forces predicted by the models were compared to experimental data. Limb forces at different stance distances were well predicted by both minimum-effort solutions. Muscle tuning directions were found to be invariant across postural configurations, similar to experimental data, but the quality of the muscle pattern predictions were not quantified and there also appeared to be discrepancies (see their Figure 8), especially for the minimum muscle effort solution (e.g., no activity predicted in biceps femoris and gracilis), matching our observations in the human arm. McKay and Ting concluded that reduced-dimension neural control mechanisms, such as muscle synergies, can achieve similar kinetics to optimal solutions, demonstrating the feasibility of muscle synergies as physiological mechanisms for the implementation of near-optimal motor solutions. In our study we could not assess kinetics predictions, as the generation of a specific force target was a constraint in the optimization. However, our analysis of muscle pattern predictions also supports the conclusion that three-dimensional forces are generated as near- or sub-optimal motor solutions by muscle synergy combinations.

The fact that the observed muscle activation patterns did not minimize muscle or synergy effort does not rule out the possibility that they minimized some other cost. The additional co-contraction inherent in the non-minimal effort solutions might be related to an increase in stiffness during the hold phase possibly due to endpoint stability maximization (Franklin and Milner, 2003). Since the task was isometric, in principle there was no need to increase endpoint stiffness to generate a target output force precisely. On the contrary, because of signal-dependent noise in force production by muscle activation, the precision would decrease with an increase in co-contraction. However, subjects had to control a moving cursor in a realistic virtual environment and they might have adopted a control strategy usually employed when required to generate a force while maintaining a freely moving endpoint, typically a tool, close to a fixed position. In those conditions an increase in stiffness associated to an increase in co-contraction would be an appropriate control strategy to achieve higher positional stability at the cost of an additional muscular effort. Thus, as suggested in recent studies, the CNS might adopt habitual rather than optimal (de Rugy et al., 2012) or locally rather globally optimal (Ganesh et al., 2010) muscle coordination strategies.

Whether muscle synergies are organized by the CNS to simplify motor control and motor learning (Giszter et al., 2007; Bizzi et al., 2008; d'Avella and Pai, 2010; Bizzi and Cheung, 2013; d'Avella and Lacquaniti, 2013) or they are results from biomechanical and task constraints (Todorov and Jordan, 2002; Kutch et al., 2008; Kutch and Valero-Cuevas, 2012) is a controversial issue (Tresch and Jarc, 2009). Evidence for muscle synergies as neural control strategies has come mainly from the low-dimensionality in the muscle patterns recorded during a variety of behaviors and task conditions and across different species (Tresch et al., 1999; d'Avella et al., 2003, 2006, 2008, 2011; Krishnamoorthy et al., 2003; Hart and Giszter, 2004; Ivanenko et al., 2004, 2005; Cheung et al., 2005; Ting and Macpherson, 2005; Overduin et al., 2008; Muceli et al., 2010; Dominici et al., 2011; Chvatal and Ting, 2013; D'Andola et al., 2013; Gentner et al., 2013), from neural recordings and stimulation (Saltiel et al., 2001; Ethier et al., 2006; Gentner and Classen, 2006; Gentner et al., 2010; Hart and Giszter, 2010; Overduin et al., 2012), and, recently, from the observation that adaptation to a perturbation of the normal mapping between muscle activity and force, simulated in a virtual environment using myoelectric control, is slower when the perturbation is not compatible with the synergies than when it is (Berger et al., 2013).

Two recent studies (Kutch et al., 2008; Kutch and Valero-Cuevas, 2012) have argued against the neural origin of the muscle synergies involved in the generation of isometric forces. In a first study, Kutch and colleagues compared the directional dependence of the covariance of the force fluctuations observed experimentally during the generation of planar isometric forces with the index finger with the directional dependence predicted by either a minimum synergy effort model of a minimum muscle effort model (Kutch et al., 2008). They argued that, if individual muscles are activated flexibly and the force they generate is affected by signal-dependent noise (Harris and Wolpert, 1998), the force generated in the direction of action of an individual muscle must show a covariance ellipse elongated in the direction of the force. In contrast, if muscles are recruited within fixed synergies, multiple muscles are always activated simultaneously and the force covariance must be on average less elongated in the direction of the target force. For isometric forces generated by the index finger on a plane, the observed force covariance directness was found to be more in agreement with the directedness predicted by minimum muscle effort than the directedness predicted by minimum synergy effort. However, we wonder whether the results of Kutch and colleagues depended on the fact that the synergies used in their calculation were not extracted from the data but generated randomly, while the directedness of the synergy model was evaluated only in three fixed directions corresponding to the peak values of the directedness of the data. We plan to test the directedness of the force covariance of three-dimensional forces generated at the hand by several arm muscles in a future study.

In a more recent study, Kutch and Valero-Cuevas studied the generation of isometric forces by actuation of the tendons of a cadaveric index finger and with a model of the human leg (Kutch and Valero-Cuevas, 2012). They argued that, if the set of all possible muscle coordination patterns that produce any single endpoint force vector are themselves a low-dimensional subset, the observed low-dimensionality of the muscle patterns could be misinterpreted as neurally-generated muscle synergies. Principal component analysis was performed on the set of all vertices of the solution set in muscle activation space for 16 planar force directions, identified with computational geometry techniques using the linear muscle-to-force mapping derived experimentally or from the model. The dimensionality of all possible coordination patterns resulted indeed lower than the number of muscles, thus, providing an assessment of the upper limit imposed by biomechanics, but, at least for the leg model, higher than the dimensionality typically observed in the data. Thus, such biomechanical limit to dimensionality should be directly compared to the dimensionality extracted from experimentally observed muscle patterns, as we also plan to do, to draw any conclusion on the neural origin of muscle synergies.

In conclusion, we have demonstrated that muscle patterns underlying the generation of three-dimensional forces can be reconstructed accurately by the combination of a small number of muscle synergies but they could not be predicted accurately by either minimization of muscle effort or synergy effort. However, the minimum synergy effort model fitted the experimental data much better than the minimum muscle effort model, suggesting that the CNS generates three-dimensional forces by sub-optimal recruitment of muscle synergies.

### Conflict of interest statement

The authors declare that the research was conducted in the absence of any commercial or financial relationships that could be construed as a potential conflict of interest.
